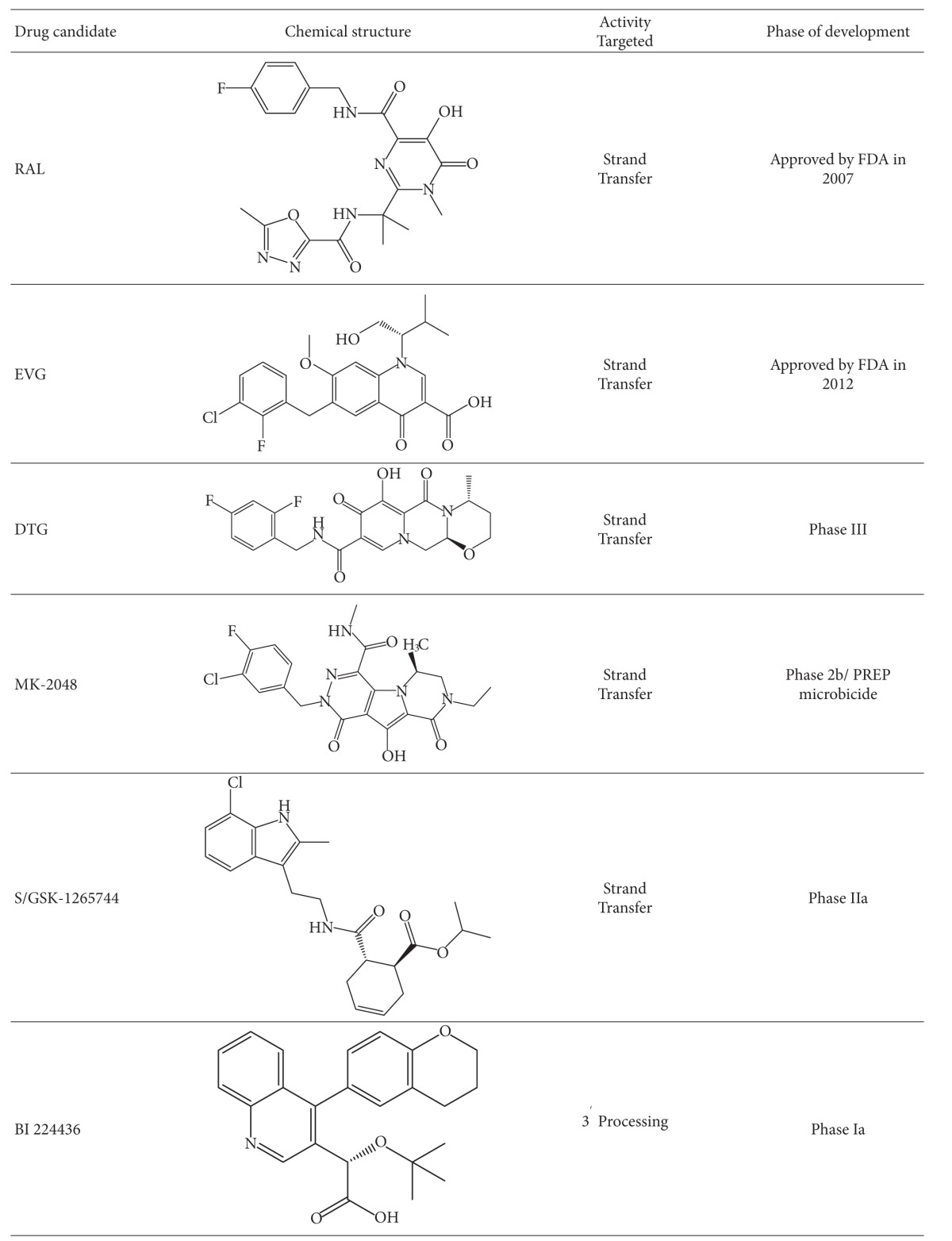# The Need for Development of New HIV-1 Reverse Transcriptase and Integrase Inhibitors in the Aftermath of Antiviral Drug Resistance

**DOI:** 10.6064/2012/238278

**Published:** 2012-12-31

**Authors:** Mark A. Wainberg

**Affiliations:** Lady Davis Institute, McGill University AIDS Centre, Jewish General Hospital, Montreal, QC, Canada H3T 1E2

## Abstract

The use of highly active antiretroviral therapy (HAART) involves combinations of drugs to achieve maximal virological response and reduce the potential for the emergence of antiviral resistance. There are two broad classes of reverse transcriptase inhibitors, the nucleoside reverse transcriptase inhibitors (NRTIs) and nonnucleoside reverse transcriptase inhibitors (NNRTIs). Since the first classes of such compounds were developed, viral resistance against them has necessitated the continuous development of novel compounds within each class. This paper considers the NRTIs and NNRTIs currently in both preclinical and clinical development or approved for second line therapy and describes the patterns of resistance associated with their use, as well as the underlying mechanisms that have been described. Due to reasons of both affordability and availability, some reverse transcriptase inhibitors with low genetic barrier are more commonly used in resource-limited settings. Their use results to the emergence of specific patterns of antiviral resistance and so may require specific actions to preserve therapeutic options for patients in such settings. More recently, the advent of integrase strand transfer inhibitors represents another major step forward toward control of HIV infection, but these compounds are also susceptible to problems of HIV drug resistance.

## 1. Introduction

Infection by the human immunodeficiency virus (HIV) is a major problem and the treatment for this condition is commonly referred to as highly active antiretroviral therapy (HAART). The latter consists of three or more HIV drugs, most commonly two nucleoside reverse transcriptase inhibitors (NRTIs) in combination with either a nonnucleoside reverse transcriptase inhibitor (NNRTI), a protease inhibitor (PI) or, more recently, an integrase inhibitor (INI) [1]. The goal of HAART is to optimally suppress HIV replication during long-term therapy and to maintain immune function [2]. Rational drug selection is essential to maximize potency, minimize side effects and cross resistance, preserve future treatment options, and increase overall duration of viral suppression (reviewed in [3]). Although numerous antiretroviral combinations may provide potent suppression of viral replication, therapeutic choices necessitate careful consideration of the potential impact of viral resistance on subsequent treatment.

Progress in antiretroviral therapy has improved HIV management and the control of the spread of regional epidemics [4]. However resistance to antiretroviral drugs is largely unavoidable, due to the error-prone nature of HIV reverse transcriptase (RT), and its lack of a proofreading function [5]. In addition, the sheer number of replication cycles occurring in an infected individual and the high rate of RT-mediated recombination events, facilitate the selection of drug resistant mutant strains of HIV [6, 7]. Furthermore, certain tissue compartments seem able to select for resistance mutations due to the presence of low drug concentrations. [8]. These mutations are located on the genes that encode antiretroviral targets such as RT, resulting in the production of RT that is different than its wild-type (wt) counterpart in both structure and function. Although this protein is still able to play its role in HIV replication, it is not inhibited as effectively as wt protein by the antiretroviral (ARV) compounds. HIV RT is a heterodimer with the polymerase and RNase H activity provided by the larger subunit p66; p66 contains defined domains, that is, a polymerase domain, a C-terminal RNase H domain, and a connection domain.

The number of mutations required for resistance to occur varies from drug to drug. Many factors determine the relative rate of resistance selection with different drugs and drug combinations, and this is reflected in the “genetic barrier” to resistance which refers to the number of mutations that must occur within a given target in order for resistance to develop against a particular drug and the speed with which this happens. Interactions between mutations, the effects of individual resistance mutations on viral replication capacity, and viral fitness all influence mutational pathways and the overall impact of resistance mutations on viral phenotype. Many different mechanisms through which HIV-1 escapes from drug pressure have been described; these mechanisms differ from one drug class to another and can even differ between drugs of the same category.

## 2. Reverse Transcriptase (RT) Inhibitors 

Two distinct classes of RT inhibitors have been described, the nucleoside reverse transcriptase inhibitors (NRTIs) and nonnucleoside reverse transcriptase inhibitors (NNRTIs). NRTIs incorporate into nascent viral DNA, resulting in DNA chain termination and blocking further extension of DNA. The NNRTIs stop HIV-1 replication by binding to the hydrophobic pocket within the p66 sub unit of the RT enzyme thus preventing it from converting viral RNA into DNA [9, 10]. NNRTIs are non-competitive inhibitors of HIV-1 RT and do not require activation. The low fidelity of HIV-1 RT, high level of HIV-1 replication and the high rate of RT mediated recombination collectively contribute to the emergence of resistance to RT inhibitors [7, 11].

### 2.1. First-Generation NRTIs

Resistance of HIV to NRTIs can occur via two distinct mechanisms. The first is discrimination, whereby the mutated viral RT can selectively avoid incorporating NRTIs in favour of natural dNTPS; this mechanism is typified by such mutations as K65R, L74V, Q151M, and M184V [12]. The second mechanism of resistance allows a mutated RT to enact the phosphorolytic excision of NRTIs from the 3′ end of the viral DNA chain that extends from the primer, a process referred to as “primer unblocking”. Examples of mutations involved in this process are those selected by zidovudine (ZDV) and stavudine (d4T), that are termed thymidine analogue mutations (TAMs), for example, M41L, D67N, K70R, L210W, T215Y/F, and K219Q/E [13, 14]. TAMs confer resistance to all NRTIs except lamivudine (3TC) and emtricitabine (FTC). Although, there is a degree of cross-resistance associated with TAMs, ultimate levels of resistance depend on the specific NRTI and the number of TAM mutations found in the viral RT [14]. 

The two distinct NRTI resistance mechanisms of discrimination and excision can also influence each other. For example, the M184V/I mutation that is selected by 3TC and FTC is a discrimination mutation but viruses that contain M184V/Iare less likely to quickly develop tams under selective pressure with such drugs as ZDV viruses containing M184V/I are also more susceptible to ZDV and d4T than wild-type viruses [14].

 HIV therapy with abacavir (ABC) leads to the selection in many cases of one to three of the following mutations: K65R, L74V, Y115F, and M184V [15]. The resistance profile most frequently observed is the combination of L74V and M184V. The K65R mutation is selected by tenofovir (TFV) and reduces susceptibility by 3 to 6-fold [16, 17]. Usually, the selection of K65R precludes the occurrence of TAMs while the presence of the latter mutations prevents the selection of K65R due to the fact that viruses that contain both K65R and TAMs are not viable. The gold standard in patients initiating therapy involves a combination of TFV/FTC or ABC/3TC together with a NNRTI [18].

### 2.2. First-Generation NNRTIs

Efavirenz (EFV) and Nevirapine (NVP) are FDA approved NNRTIs and have become the cornerstone of therapy within both developed and under-developed countries. However, the low genetic barrier to resistance of these first-generation NNRTIs serves as a major limitation for prolonged antiretroviral therapy and sequential use of inhibitors of this class [19–21]. Notably, a single amino acid substitution in the RT enzyme is often adequate to yield-high-level clinically relevant resistance. Additionally, high-level cross-resistance among first-generation NNRTIs has been reported [19] and this can also have an impact by decreasing virologic response in patients with transmitted resistance [22]. The prevalence rate of transmitted antiretroviral drug resistance in treatment-naïve patients with HIV-1 has been estimated to be 5–15% for resistance mutations to at least one antiretroviral class [23]. In this US drug resistance survey, the NNRTI class showed the highest prevalence at a rate of 6.9% compared to NRTIs and PIs at 3.6% and 2.4%, respectively. The increasing use of NNRTIs in clinical practice and the fact that NNRTI resistance mutations do not severely impair viral replication capacity but remain part of the dominant viral variant may explain the high prevalence of NNRTI resistance [24].

The binding pocket for NNRTIs is located largely in the p66 subunit of RT and consists of the following residues; 95, 100, 101, 103, 106, 108, 179, 181, 188, 190, 227, 229, 234, 236, and 318 [10, 25]. Some residues from p51, such as 138, also contribute to the NNRTI binding. Mutations conferring NNRTI resistance generally affect interactions between the inhibitor and the enzyme. NNRTI resistance mutations can inhibit drug binding through at least three mechanisms: (1) They can block the entry of inhibitor into the NNRTI binding pocket (e.g., K103N); (2) They can affect contacts between the inhibitor and residues that line the NNRTI binding pocket (e.g., Y181C); or (3) they can alter the conformation or size of the NNRTI binding pocket so that it becomes less specific for the inhibitor (e.g., Y188L) [24]. Some resistance mutations can affect the binding of NNRTIs through several mechanisms [25].

### 2.3. Newer NRTIs

#### 2.3.1. Elvucitabine

This compound (Table 1) is a novel NRTI currently in late phase II study and was developed by Achillion Pharmaceuticals. In an *in vitro* selection study, only two amino acid substitutions, M184I and D237E, were identified in the resultant variant [26]. The double mutation conferred moderate resistance to elvucitabine (about 10 fold) and cross-resistance to lamivudine but not to other nucleoside inhibitors tested. Elvucitabine has also demonstrated potent antiviral activity in HIV-infected patients with resistance to 3TC and other NRTIs. The drug has good oral bioavailability and an intracellular half-life of > 24 hours [27].

#### 2.3.2. Apricitabine (ATC)

This compound (Table 1) is a novel deoxycytidine NRTI currently in clinical development for the treatment of HIV infection. *In vitro* selection for resistance with ATC selected for M184V, V75I and K65R [28]. The resulting mutants from this selection conferred low-level resistance of less than 4 fold. Others showed that continuous passage of HIV-1 already containing M184V, K65R or combinations of M41L, M184V, and T215Y did not result in any additional mutations [29]. ATC has shown antiviral activity *in vitro* against HIV-1 strains and clinical isolates with NRTI mutations that include M184V, L74V, and TAMs [30]. It has activity in treatment-naïve and treatment-experienced HIV-1 infected patients with M184V and up to 5 TAMs. Resistance to ATC was reported to be slow to develop *in vitro,* and there is little evidence of the development of resistance to this drug in infected patients [28–30]. 

#### 2.3.3. 4′-Ethynyl-2-Fluoro-2′-Deoxyadenosine (EFdA)

This compound (Table 1) is a new NRTI, now in preclinical development, that retains the 3′-OH group and has excellent antiviral properties (EC_50_ of 0.07 nM) against wt virus [31, 32] compared to approved NRTIs with an EC_50_ range of 17 to 89 nM [33]. This robust antiviral activity is due to a mechanism of action that is different from approved NRTIs. Notably EFdA-TP acts by binding through its 3′-primer terminus to RT; the addition of subsequent nucleotides is than prevented by blocking the translocation of the primer strand on the viral polymerase [34]. Thus, EFdA-TP is termed a “translocation-defective reverse transcriptase inhibitor (TDRTI)”. Modeling studies have confirmed the binding of EFdA-TP to a hydrophobic pocket of RT residues Ala-114, Tyr-115, Phe-160, and Met-184 and the aliphatic chain of Asp-185 [34].

*In vitro* pre-steady-state kinetics studies to determine toxicity showed that EFdA-TP is a poor human mitochondrial DNA polymerase^*γ*^ (Pol^*γ*^) substrate, suggesting that Pol^*γ*^-mediated toxicity might be minimal [35]. Resistant variants including those containing the K65R/L74V/Q151M complex did not affect the susceptibility of this compound while the HIV-1 clone containing M184V alone conferred low to moderate level resistance to EFdA-TP [32]. *In vitro*, a parental compound of EFdA-TP, 2′-deoxy-4′-C-ethynyl-adenosine (EdA) selected for resistant variants after 58 passages with novel combination of mutations, I142V/T165R/M184V [32]. Site directed mutagenesis of clones containing the mutations showed that either I142V or T165R alone did not affect the antiviral activity of EFdA-TP while M184V alone or in combination with I142V or T165R demonstrated moderate resistance to EFdA-TP. The triple mutant I142V/T165R/M184V had the highest resistance among relevant clones that were tested [32].

*GS-9131*. This compound (Table 1) is a prodrug of the nucleotide reverse transcriptase analogue GS-9148, which belongs to the same family as TFV. GS-9131 demonstrated potent antiviral activity against a variety of HIV-1 subtypes, that is, EC_50_ of 37 nM [36]. *In vitro*, the parent drug (GS-9148) caused only low-level cytotoxicity compared to TFV (12). GS-9131 also demonstrated synergy in combination with other antiretrovirals as well as potent antiviral activity against multi-NRTI resistant strains, including K65R, M184V, and L74V, and only a minimal increase in EC_50_ in regard to viruses carrying four or more TAMs [36]. The use of GS-9131 was shown to result in 76–290 times more of the di-GS-9148 compared to GS-9148 [37]. 

*GS-7340*. This compound (Table 1) is a prodrug of TFV that exhibits anti-HIV activity and that possesses a favorable resistance profile. The EC_50_ of GS-7340 against HIV-1 in MT-2 cells was 0.005 *μ*M compared to 5 *μ*M for the parent drug TFV [38]. In HIV-I infected patients GS-7340 demonstrated enhanced antiviral activity, with no TFV mutations identified, and yielded higher intracellular concentrations of TFV-diphosphate than TFV [39].

*CMX157*. This compound (Table 1) is a lipid moiety prodrug of TFV that has activity against wt viruses of major HIV-1 subtypes with EC_50_s ranging from 0.2 to 7.2 nM [33]. In contrast TFV has EC_50_s against HIV-1 group M and O that range between 500–2200 nM in PBMCs [40]. In an *in vitro* study, CMX157 demonstrated potent activity against NRTI-resistant strains, including multidrug-resistant viruses against which it showed >300-fold activity compared to TFV [33]. The higher potency and lower EC_50_ of CMX157 is due to better cellular uptake than TFV and the fact that it is not a substrate for organic anion transporters. This permits it to maintain high concentrations cells, in contrast to TFV that is actively metabolized by organic anion transporters, leading to decreased intrracellular concentrations [41]. *In vitro* and preclinical studies in rats showed that CMX157 also has a favorable cytotoxicity profile in PBMCs and that it does not lead to nephrotoxicity as has been reported for TFV [33, 42]. No information is available on the selection of resistance mutations with CMX157 at this time. 

*Amdoxovir (AMDX)*. This compound is a prodrug of *β*-D-dioxolane guanosine (DXG) that is currently in phase II clinical trials (Table 1). *In vitro* phenotypic analyses have shown that DXG is effective against HIV-1 variants that are resistant to lamivudine and emtricitabine (M184V/I) as well as against viruses that contain TAMs, while selection studies in MT-2 cells resulted in the appearance of K65R and L74V [43, 44]. The combination of AMDX together with zidovudine (ZDV) in HIV-1 infected patients was shown to be synergistic, resulting in reduced viral loads [45]. AMDX thus represents a new NRTI that possesses potent antiviral activity against NRTI-resistant viruses. 

*Festinavir (OBP-601)*. This compound (Table 1) is a new NRTI in the same family as stavudine (d4T) but that has an improved safety profile. *In vitro,* it shows potent antiviral activity against wt HIV-1 of multiple subtypes with EC_50_s ranging from 0.76–5.8 *μ*M, compared to 1.57 to 6.06 *μ*M for d4T [46]. In a phenotypic susceptibility assay, viruses carrying the K65R and Q151M resistance mutations were shown to be hypersusceptible to this compound. In contrast, a slightly decreased antiviral response was observed against viruses carrying either TAMs or TAMs together with K103N and M184V [46]. More importantly, a strong synergistic effect of OBP-601 with several approved NRTIs and NNRTIs was observed against resistant and wt HIV-1 isolates [46].

### 2.4. Newer NNRTIs

#### 2.4.1. Etravirine (ETR)

This compound (Table 2), formerly known as TMC125, is a diarylpyrimidine (DAPY)-based NNRTI that possesses potent antiviral activity against both wt HIV-1 of multiple subtypes as well as against some viruses resistant to first-generation NNRTIs [47, 48]. Specifically, ETR retains full activity against viruses containing the most prevalent NNRTI mutation K103N [47]. *In vitro*, ETR is more difficult to generate resistance against compared to initial NNRTIs [48]. Clinical studies have shown that ETR together with potent background regimens, that include NRTIs, as well as integrase and protease inhibitors, significantly decreased viral loads in patients with resistance against older NNRTIs and some PIs [49–51]. *In vitro* selection studies and both the DUET-1 and the DUET-2 clinical trials have now identified 20 ETR resistance-associated mutations (RAMs) (V90I, A98G, L100I, K101E/H/P, V106I, E138A/K/G/Q, V179D/F/T, Y181C/I/V, G190A/S, and M230L) and have permitted the assignment of a weighted score for each mutation [52–54]. Of these ETR RAMs, three or more are required for high level resistance to occur, thus demonstrating a high genetic barrier to resistance compared to older NNRTIs. The structure of ETR allows it to bind to the RT enzyme, such that changes in the NNRTI-binding pocket do not compromise binding and, thus, activity is maintained. This allows ETR to reorient itself and provides alternative binding conformations when mutations in the binding pocket occur [55]. Because of its unique characteristics, ETR is the only NNRTI approved for treatment of experienced patients who have previously received other NNRTIs. 

Few studies have prospectively studied the efficacy of ETR in combination with other background regimens in NNRTI experienced patients [56, 57]. In the phase III DUET-1 and DUET-2 studies, 57% of patients in the ETR arm versus 36% in the placebo had a viral load <50 copies/mL after 96 weeks of treatment [56]. 

In the DUET-1 and DUET-2 clinical studies, it was found that patients who experienced virologic failure had greatest numbers of ETR resistance mutations at baseline than patients who had treatment success. Second, patients who experienced virologic failure were often found to have received background regimens that were less potent than the drugs given to patients who did not fail therapy [58]. In this study, the V179F, V179I, and Y181C mutations in RT were commonly associated with treatgment failure alongside changes at positions K101 and E138 [58]. The authors concluded that these mutations usually emerged in a background of other multiple NNRTI mutations and were in most cases associated with a decrease in phenotypic sensitivity to ETR. 

A subsequent subanalysis of the DUET trial studied the impact of background regimen on virologic response to ETR, and the authors further confirmed that a higher virologic response rate was observed in patients who demonstrated an increased activity of the background regimen, with the highest responses being achieved in patients who used more than two active agents in addition to ETR [59]. In the TMC125-C227 (ETR) trial, ETR was inferior to a protease inhibitor (PI) in PI-naïve patients with a history of previous NNRTI failure [60]. In a post-hoc analysis of baseline resistance data for the DUET trials, a diminished virological response was observed in patients who possessed the following characteristics at baseline; the presence of Y181C, a baseline ETR fold change of ≥10, and a higher number of ETR resistance mutations [60]. In another study, the authors studied 42 NNRTI treatment-experienced patients for 6 months on an ETR containing regimen [57]. At failure, 12 of 42 patients developed at least one new NNRTI mutation. The most frequently selected mutations included V179I, Y181C and V179F among others. 

Several studies have also researched the theoretical potential of ETR, based on the resistance patterns of patients who previously failed NNRTI therapy and accumulated ETR RAMs. These studies have observed a prevalence of more than three ETR RAMs among viral isolates from patients experiencing NNRTI treatment failure, ranging from 4.6% to 10%, while the prevalence of isolates with single ETR RAMs was 17.4% to 35.9% [61–64]. These studies concluded that there is a low prevalence of ETR resistance at baseline and that patients with prior failure to NNRTIs could potentially benefit from ETR rescue therapy. However, these analyses focused on patients in developed countries that have full access to the most potent antiretroviral drugs and patients are constantly monitored for the development of resistance and viral load. 

It should be noted that patients in countries with limited resources developed resistance faster due to a lack of potent antiretroviral drugs and drug resistance testing. Some studies in resource-limited settings have observed a high prevalence of NNRTI resistance mutations associated with ETR resistance among patients failing an NNRTI containing regimen [65–67]. Using NVP in the failing regimen was associated with intermediate and reduced response to ETR while use of EFV and coadministration of 3TC reduced the risk of ETR resistance [65]. The authors concluded that the frequent occurrence of NNRTI mutations in resource-limited settings in which drug resistance testing is rare might compromise the continuous use of ETR and also its use in second line therapy. The Y181C mutation, associated with NVP therapy, has been reported with a high prevalence in NNRTI experienced patients and shown to decrease susceptibility to ETR [61, 65, 68, 69]. This demonstrates cross-resistance of both NVP and ETR as has been confirmed [53]. Thus, the widespread use of NVP in resource-limited settings without resistance testing casts doubt as to whether ETR could be effective in NNRTI experienced patients in poorer countries. One of these studies suggested that ETR should be avoided in salvage regimens in the setting of first-line NVP failure where drug resistance testing is not performed [68]. Another study analyzed the prevalence of minority variants in treatment-naïve and NNRTI experienced patients by ultradeep pyrosequencing: while such variants were not identified in any of 13 drug-naive patients, it was shown that 7 of 20 patients who had failed an NNRTI-containing regimen possessed minority variants as well as ETR associated-NNRTI resistance mutations [70]. This suggests that minority variants in NNRTI experienced patients may lead to virologic failure and to decreased ETR activity [71]. 

Tissue culture selection and clinical trials results with ETR have identified amino acid substitutions at position 138 in RT [53, 54]. Mutations at position 138 are not associated with resistance to older NNRTIs. Although these mutations conferred only low-level resistance to ETR, E138K is also associated with resistance to most newer NNRTIs (Table 2); its emergence may also facilitate the development of additional ETR mutations [53]. The connection domain mutation N348I has been identified and implicated in reduced susceptibility to NVP and EFV as well as to the nucleoside analogue ZDV [72]. Two independent studies have assessed and confirmed that this mutation also decreases susceptibility to ETR, either alone or in combination [73, 74]. This effect was reversed when M184V was coexpressed with N348I. Additional connection domain mutations found to be associated with impaired susceptibility to ETR were T369I and E399G. 

Several independent genotypic scores have been established to predict ETR resistance. The first, developed by Janssen, has been correlated with treatment response in the DUET studies and, when combined with *in vitro* selection studies, identified 17 (recently updated to 20) ETR RAMs [52, 54]. In this analysis, any three or more of these mutations were required to cause resistance to ETR. A second score by Monogram is based on a correlation of phenotype and genotype results of 4,923 samples containing at least one NNRTI mutation [75]. The Monogram genotypic score identified 30 mutations associated with ETR resistance with the weighted score for each mutation being slightly higher than the 20 mutation-based Janssen score. The two scores yield similar results.

#### 2.4.2. Rilpivirine (RPV) (TMC278)

This drug (Table 2) is another DAPY compound that was recently approved for treatment of NNRTI-naïve patients. The structure and binding of RPV in the NNRTI binding pocket is similar to that of ETR, which allows reorientation of both compounds within RT. *In vitro*, RPV possesses subnanomolar activity against wt HIV-1 of multiple subtypes and shows antiviral activity against viruses containing many NNRTI resistance-associated mutations [76]. NNRTI RAMs emerging in culture under RPV selective pressure included combinations of V90I, L100I, K101E, V106A/I, V108I, E138G/K/Q/R, V179F/I, Y181C/I, V189I, G190E, H221Y, F227C, and M230I/L. The resistance profile and genetic barrier to the development of resistance of RPV are comparable to those ofETR. High-resolution crystal structures ofRPV incomplex withHIV-1 RT reveal that the cyanovinyl group of TMC278 is positioned in a hydrophobic tunnel connecting the NNRTI-binding pocket to the nucleic acid-binding cleft [77]. Both RPV and ETR exhibit similar flexibility in adapting to resistance mutations. In the ECHO and THRIVE phase 3 trials [78, 79], resistance analysis showed a slightly higher proportion of treatment failures in the RPV arm compared to the EFV arm. The most frequent NNRTI mutation in the RPV arm was E138K in addition to mutations such as Y181C, K101E, H221Y, V901, E138Q, and V189I [80]. Also, the proportion of NRTI mutations that emerged in the study was higher in the RPV arm than the EFV arm. The NRTI mutations selected included Y115F, M184I/V, and K219E. 

Recently, a survey was made of 15 mutations (K101E/P, E138A/G/K/Q/R, V179L, Y181C/I/V, H221Y, F227C, and M230I/L) associated with decreased susceptibility to RPV [81]. These mutations have been described based on *in vitro* studies and in patients in whom RPV was failing. The quantitative impact of each of these mutations on RPV resistance may differ. 

#### 2.4.3. Dapivirine (TMC 120)

This compound (Table 2) is another DAPY compound that can accommodate some mutations within the NNRTI binding site without significant loss of activity [20, 82]. TMC 120 has shown potent antiviral activity against both wt and NNRTI-resistant HIV-1 strains [83, 84]. In 2004, Janssen officially licensed the further development of TMC 120 for use as a vaginal microbicide to the International Partnership for Microbicides (IPM) to help prevent HIV-1 sexual transmission. 

Both phase I and II studies have shown that TMC 120 was widely distributed through the lower genital tract with low systemic absorption when administered as a vaginal gel formulation for up to 42 days [85, 86]. The gel was safe and well tolerated. *In vitro* selection studies have identified drug resistance mutations in the presence of TMC 120, notably L100I, K101E, V108I, E138K/Q, V179M/E, Y181C, and F227Y [87, 88]. Most of these TMC 120 resistance-associated mutations occur at exactly the same position as many of the mutations associated with ETR and RPV resistance [52, 53, 76]. However, in one of these studies, it was shown that suboptimal concentrations of TMC120 alone facilitated the emergence of common NNRTI resistance mutations while suboptimal concentrations of TMC120 plus tenofovir (TFV) gave rise to fewer mutations [88]. Due to the likelihood of transmitted resistant strains in HIV-1 infected individuals, resistance mutations might impact the ability of a single drug in preventing HIV-1 as a microbicide. Using a combination of antiviral drugs of different classes may be useful. Another study showed in an *in vitro* model that using TMC 120 in combination with TFV as a microbicide was more potent and exhibited synergy in comparison with the use of either drug on its own [84]. 

#### 2.4.4. Lersivirine

This compound (Table 2) is a new NNRTI belonging to the pyrazole family and is being developed by Pfizer. An *in vitro* resistance study Lersivirine selected for the amino acid substitutions; V108I, E138K, V179D, F227L, and M230I [89]. In a phase II b trial, were reported better responses in patients with EFV compared to Lersivirine, that is, 86% versus 79%, respectively, [90]. Among patients who failed lersivirine, the mutations identified included K101E, V106M, V108I, H221Y, Y188H, F227C/L, and L234I among others [90].

#### 2.4.5. RDEA806

This compound (Table 2) is a novel NNRTI being developed by Ardea Biosciences. In genotypic and phenotypic analyses of mutant viruses selected by RDEA806, the K104E, E138K, T240I, V179D, and F227L substitutions were identified [91]. Phenotypic analysis of these mutations demonstrated that RDEA806 requires at least 3 mutations for greater than 10 fold loss of susceptibility. In a phase 2a trial, RDEA806 was well tolerated and exhibited robust antiviral activity with no phenotypic or genotypic alterations [92].

## 3. Integrase Inhibitors

### 3.1. Early Integrase Inhibitors

The integrase (IN) enzyme of HIV is pivotal in the viral replication cycle as it catalyzes the insertion of the reverse transcribed viral genome into host chromatin. Integrase catalyzes two distinct steps: 3′ processing and strand transfer. During 3′ processing, integrase excises a dinucleotide from the 3′ terminus of viral cDNA. 3′ processed viral DNA is then covalently linked to host DNA during strand transfer [93]. This unique process has always been considered a viable drug target that several early studies attempted to exploit [94]. Early integrase inhibitors (INIs) included peptides [95, 96], nucleotides [97], DNA complexes [98] as well as small molecules derived either from natural products [97] or by rational drug design strategies [96, 99]. Even though some of these compounds advanced into preclinical trials, further clinical development was always curtailed due to *in vivo* toxicity and/or nonspecific off-target effects. More detailed reviews on the development of early INIs have appeared in the literature [94, 96, 100]. A fuller description of the mechanism of action of integrase inhibitors is found below in the section on Resistance.

In order for an inhibitor to be considered useful as an antiviral in combination therapy for HIV, selectivity (such as for IN) which is distinct from effects on other targets (such as RT and protease) needs to be proven. The 4-aryl-2,4-diketobutanoic acid inhibitors containing a distinct diketo acid moiety (DKA) were identified in 2000 by Merck investigators from a screen of 250 000 compounds, and for a time were the only biologically validated INIs [101]. Their antiviral activity in cell culture was mitigated by the development of resistance mutations in the IN protein, thereby confirming their mode of action [101]. These compounds, exemplified by L-731988 [102], were found to inhibit strand transfer with much higher potency (IC_50_ = 80 nM) than 3′ prime processing (6 *μ*M) [101], and they were thus referred to as strand transfer inhibitors (STIs). IN, like most phosphotransferase enzymes, requires two divalent cations bound at the active site for activity; Mg^2+^ is likely used *in vivo*, although Mn^2+^ is used in some *in vitro* assays [103]. Most STIs that have been described, including DKA compounds, inhibit IN by chelation of bound cations in a dose-dependent manner [104]. The crystal structure [105] of IN bound to the prototype DKA, 1-(5-chloroindol-3-yl)-3-hydroxy-3-(2H-tetrazol-5-yl)-propenone (5-CITEP) [106], provided structural evidence for the DKA-specific mode of action. The compound termed 5-CITEP was found to bind in proximity to the evolutionarily conserved D64 D116 E152 motif of IN also providing valuable structural confirmation of the IN active site [105]. Subsequent variations of DKAs based on the 5-CITEP backbone led to increased potency, specificity, tolerability, and bioavailability. This, in turn, led to the first clinically tested INI (S-1360) [106]. Despite an initially good pharmacological and pharmacokinetic profile in animal models, S-1360 in initial human trials was found to be rapidly cleared through glucoronidation [107] and its development was not pursued.

### 3.2. Clinically Approved Integrase Inhibitors

#### 3.2.1. Raltegravir

The study and optimization of lead compounds including L-31988 and L-870812 by Merck pharmaceuticals led to the development of Raltegravir (Issentris, MK-0518) (Table 1), which in 2007 became the first INI (and currently only) approved for treatment in both antiretroviral (ARV) naive and treatment-experienced patients [108]. Raltegravir (RAL) [108] was shown in multiple trials, such as BENCHMRK, to achieve efficient viral load suppression in ARV-experienced patients when included in an optimized background ARV regimen [109]. In the BENCHMRK trials, 57% of patients achieved plasma levels of HIV-1 RNA <50 copies/mL after 97 weeks of therapy, whereas only 26% of the placebo group, treated with only optimized background regimen (OBR) drugs, achieved viral suppression. The efficacy of RAL relative to other ARVs has been modeled in cell culture and has been shown to be due to the activity of INIs at later stages in the viral replication cycle than either viral entry or reverse transcription inhibitors: they are therefore able to inhibit replication in a larger proportion of productively infected cells [110]. In another study of patients with multi-drug resistant viruses with a median ARV treatment experience of 9 years, a RAL-containing regimen yielded higher viral load suppression than a regimen containing placebo when combined with OBR [111]. RAL has a favorable toxicity profile and does not appear to have a high propensity for clinically relevant drug-drug interactions [103], perhaps because of its induction of the glucuronidation enzyme UGT1A1 responsible for RAL elimination [112]. Interactions with drugs such as rifampin may lead to modest decreases in RAL half life and blood concentration after 12 hrs (C12 hr). Predictably, other protease inhibitors, such as atazanavir, have been shown to exert modest but not clinically relevant extension of C12 hr levels for RAL. RAL has been shown to have high bioavailability and is dosed twice daily at 400 mg/mL due to its C12 hr of 142 nM [113]. Studies to simplify RAL dosage to 800 mg once daily, boosted or unboosted by the protease inhibitor atazanavir were not as successful as had been hoped [114–116]. 

In spite of the high effectiveness of RAL for first line and salvage therapy, resistance mutations can reduce the susceptibility of virus to INIs. The occurrence of single point mutations that confer high level resistance (fold change FC > 5) to INIs have shown that RAL has a modest genetic barrier to resistance development. To date, three major resistance pathways involving nonpolymorphic residues have been extensively described and characterized for RAL; E92QV/N155H, T97A/Y143CHR, and G140CS/Q148HKR [117, 118]. Although these three pathways have been shown to arise separately, some recent reports suggest that they may be linked. The G140S/C and E92Q/V mutations by themselves impart greater than 5–10 fold resistance to RAL [119], but usually appear only after the N155H and Q148HKR mutations [120], leading to FC > 100 for the combined mutations. In addition to these major resistance mutations, several polymorphic and nonpolymorphic residues have been identified that impart <5-fold resistance to RAL. Some of these, such as T66I/L, have been shown to act synergistically with pre-existing major resistance mutations [121]. All major INI resistance mutations have a major impact on both IN activity and viral replicative capacity [122]. The result is swift reversion to wild-type virus in patients soon after therapy with INIs is withdrawn [123]. 

It has been suggested that patients without a history of nucleoside reverse transcriptase inhibitor (NRTI)-associated resistance may have an increased barrier for occurrence of resistance to RAL compared to patients with resistance to nonnucleoside reverse transcriptase inhibitors (NNRTIs) such as nevirapine (NVP) and efavirenz (EFV) [124]. Most reported virologic failures due to RAL resistance mutations have occurred in patients harbouring NRTI resistant viruses or in patients at increased risk of virologic failure [125]. This was highlighted in the SWITCHMRK1 and 2 phase III trials in patients undergoing salvage therapy with lopinavir (LPV), a protease inhibitor (PI), and who switched from LPV to RAL, despite having undetectable viremia. The results showed that 84.4% of those who switched to RAL (*n* = 353) maintained undetectable levels of viremia compared to >90% in the arm who did not switch (*n* = 354). Thus, this study failed to establish noninferiority of RAL to LPV in the treatment of ARV-experienced HIV-positive individuals with undetectable viremia [125]. Of the 11 patients who experienced virologic failure with HIV-1 RNA levels >400 copies/mL, 8 harboured RAL-resistance substitutions within the integrase gene [126]. 

#### 3.2.2. Elvitegravir (GS-9137) (EVG)

This compound (Table 3) is not a DKA but a mono-keto acid resulting from early modification of the DKA motif by the Japan Tobacco Company [127]. This work resulted in a group of 4-quinolone-3-glyoxylic acids, all of which had a single pair of coplanar ketone and carboxylic groups and retained high specificity for and efficacy against the strand transfer reaction similar to DKA compounds [128]. EVG, now being developed by Gilead Sciences, has been shown to have an *in vitro *IC_50_ of 7 nM against IN and an antiviral EC_90_ of 1.7 nM when assayed in the presence of normal human serum (NHS) [129]. EVG displayed ~30% bioavailability in dogs and rats with maximal plasma concentrations being achieved in 0.5–1 hr post dose [129]. In clinical trials, EVG was found to be well tolerated and efficacious [130] Pharmacokinetic boosting with ritonavir (RTV) was found to result in improved dosing [131].

CYP3A4/5 is a cytochrome p450 enzyme that is the primary metabolizing enzyme for EVG, followed by glucoronidation by UGT1A1/3 [131]. Thus, the bioavailability and clearance of EVG was found to be favored when EVG was dosed in combination with CYP3A4/5 inhibitors [103, 131, 132]. The CYP3A4/5 inhibitor, ritonavir, was found to cause a ~20-fold increase in area under the curve (AUC) and to extend elimination half-life from three to ten hours [133]. In a phase II trial of ARV-naive patients (*n* = 48) starting initial therapy on an OBR of tenofovir/emtricitabine (TDF/FTC), coadministration of EVG with a novel pharmacokinetic booster, cobicistat, in a single tablet formulation resulted in undetectable viremia in 90% of patients after 48 weeks compared to 83% of patients who received TDF/FTC/EFV [134], In a Phase IIb study, RTV-boosted EVG was noninferior to the RTV-boosted PIs darunavir (DRV) and tipranavir (TPV), when these other drugs were also used in combination therapy [135].

A potential disadvantage to the clinical use of EVG, despite it being a once-daily drug, may be that it shares a moderate genetic barrier to INI resistance with RAL and that extensive cross-resistance exists between the two compounds. The RAL signature mutations N155H, Q148H/R/K, and G140A/C/S, as well as associated accessory mutations, were selected by EVG in culture [136] and in patients [125, 137]. This precludes the use of EVG to treat most RAL-resistant viruses. The only major RAL-associated mutations not selected by EVG were Y143C/R/H and subsequent studies showed that viruses containing Y143C/R/H, remained susceptible to EVG [138]. In addition to RAL-associated resistance mutations, EVG selected for other mutational pathways. T66I did not confer high-level resistance to RAL [136], but conferred >10-fold resistance to EVG, while a T66R mutation conferred >10-fold resistance to RAL and >80-fold resistance to EVG [139, 140]. The T66I mutation is associated with a series of accessory mutations, including F121Y, S153Y and R263K; the latter two have not been associated with RAL resistance [141]. A F121Y mutation has been selected with RAL and confers high-level resistance to this compound, but has not yet been identified in the clinic [139]. Other clinically selected EVG mutations are S147G, which confers >8-fold resistance to EVG but does not affect susceptibility to RAL [139]. Other *in vitro *EVG selections resulted in several high resistance mutations that have yet to be clinically validated such as P145S, Q146P, and V151A/L [139]. The V151L mutation confers *≈*8-fold cross-resistance to RAL and has been identified in a single patient treated with this drug [142]. The use of EVG has recently been approved by the Food and Drug Administration of the US as part of a coformulation that also includes FTC, TDF, and a pharmalogic enhancer of EVG termed cobicistat.

### 3.3. Newer Integrase Inhibitors

#### 3.3.1. MK-2048

The low-to-moderate genetic barrier of resistance for the first generation INIs has led to efforts to produce second generation INSTIs with activity against RAL-resistant viruses. Optimization of tricyclic 10-hydroxy-7,8-dihydropyrazinopyrrolopyrazine-1,9-dione compounds led to the development of MK-2048 [143] (Table 3) that demonstrated a EC_95_ < 50 nM when assayed in 50% human serum and possessed a favourable pharmokinetic profile in dogs and rats [144]. MK-2048 was subsequently shown in tissue culture and biochemical assays to be effective against RAL- and EVG-resistant viruses [143–147], with only slightly diminished effectiveness against viruses containing at least two of the following mutations: E138K, G140S, Q148R [143–147]. Selection studies in culture with MK-2048 did not select for previously recognized mutations but instead selected a novel substitution at position G118R, that, in concert with E138K, conferred *≈*8-fold resistance to MK-2048 [148]. Despite its favourable resistance profile, MK-2048 has a poor pharmacokinetic profile in humans and its clinical development has been arrested. However, it has rational as a candidate microbicide for prevention of HIV infection [149]. It continues to be studied as a prototype second generation INI and has also recently shown effectiveness in the treatment of HTLV-1 in culture without causing significant tissue culture toxicity [150]. 

#### 3.3.2. Dolutegravir (DTG) (S/GSK 1349572)

This compound (Table 3) that was discovered at Shionogi Pharmaceuticals in Japan and is now being developed by a Shiniogi-ViiV Healthcare-GlaxoSmithKline joint venture is currently in phase III clinical trials [151, 152]. DTG is a promising HIV INI candidate that specifically inhibits the strand transfer reaction with recombinant purified integrase [152]. Inhibition of the integrase strand transfer reaction by DTG was later confirmed in studies with live virus, that demonstrated an accumulation of 2-LTR circles in treated cells at DTG concentrations < 1000-fold of those that caused cell toxicity [153, 154]. DTG also demonstrated efficacy against most viral clones resistant to RAL and EVG and against clinical isolates of HIV-1 and HIV-2, although some viruses containing E138K, G140S or R148R mutations possessed diminished susceptibility to DTG [152, 155–157]. Double mutants containing combinations of E138K, G140S and R148R had a fold change >10 for DTG, but this was favourable compared to RAL and EVG, which yielded a FC of >330 and >140, respectively. *In vitro *combination antiviral studies showed that DTG did not increase toxicity when used in combination, but had a synergistic effect with each of EFV, NVP, stavudine, abacavir, LPV, amprenavir, and enfurvitide as well as an additive effect in combination with maraviroc. The HBV drug adefovir and the HCV drug ribavirin had no effect on the efficacy of DTG [157], potentially allowing for its use in treating coinfections caused by these various agents. 

DTG has a pharmacokinetic profile that allows once-daily dosing without pharmacokinetic boosting. This is based on a long unboosted half-life (13–15 hr) with trough levels of DTG being much higher than the *in vitro *IC_90_ [158]. The side-effects of DTG in HIV-infected volunteers were similar to those of placebo in early stage clinical trials (phase I) [158]. 

In addition, phase IIa randomized double blind trials provided vital evidence of the anti-HIV effect and potency of DTG [159, 160]. For example, 35 ARV-experienced INI-naïve patients, who were not receiving therapy, and whose plasma HIV-1 RNA levels ranged from 3.85–5.54 log copies/mL, received once-daily doses of 2 mg, 10 mg, or 50 mg of DTG or placebo for 10 days. More than 90% of patients who received DTG, irrespective of dose, had a decline in viral load to <400 copies/mL while 70% of patients in the 50 mg arm achieved undetectable viremia. In contrast, the placebo group showed an average increase in viremia. No serious adverse effects were reported in this trial, with headaches and pharyngolaryngeal pain being the most commonly reported side effects [159]. 

The SPRING-1 double blind dose-ranging phase-II trials studied 205 ARV-naïve HIV-positive participants, with CD4^+^ cells >200 cells/mm^3^ and HIV-1 RNA >1000 copies/mL, who were treated once-daily with DTG (*n* = 155) at 10 mg, 25 mg, or 50 mg doses or 600 mg EFV (*n* = 50) combined with background therapy of abacavir/3TC or TDF/FTC [161]. Over 90% of all participants in the DTG arm had undetectable viremia after 24 weeks of treatment, establishing the noninferiority of DTG to EFV in an NRTI/NNRTI background and also showing that DTG was at least as safe as EFV. 

Until now, no primary INI resistance mutations have been reported for DTG either in culture or in the clinic. Tissue culture selection studies over 112 weeks identified, in order of appearance, viruses harboring T124S/S153F, T124A/S153Y, L101I/T124A/S153F, and S153Y by week 84. Although these mutations persisted throughout serial passaging, they did not confer high-level resistance to DTG [157]. Position 124 of IN is modestly polymorphic and S153F/Y had previously been described in EVG selection studies [162]. Despite an apparently high genetic barrier for resistance, selection studies involving DTG are ongoing, and reports that a R263K mutation in IN may confer modest *≈*3-fold resistance to DTG have been presented at conferences [163]. It has been suggested that DTG enjoys a high barrier for resistance due to a higher affinity of DTG for IN compared to RAL and EVG [164]. This hypothesis has been previously suggested for MK-2048, which also has a relatively high barrier for resistance, as it also has high affinity (*t*_1/2_ = 32 h) for IN compared to RAL (*t*_1/2_ ≥ 7.3 h) [165]. Cell-culture assays also showed that DTG exhibited tighter binding and had a longer dissociative half-life from IN than either RAL or EVG [165]. This may lead to a situation in which considerations of adherence may be less critical for DTG than for other drugs.

Work has shown that an inverse relationship existed between the half-life of binding and the inhibitory potential of INIs when the binding half-life *t*_1/2_ was below 4 h. A FC in regard to drug resistance >3, relative to wild-type, was observed when the *t*_1/2_ dropped below 1 h [164]. In these assays, the *t*_1/2_ of DTG, RAL and EVG were 71 h, 8.8 h and 2.7 h, respectively. The fact that RAL and EVG have a shorter *t*_1/2_ than DTG suggests that resistance mutations that affect binding of RAL and EVG might also be more likely to compromise antiviral potency. As an example, the Y143CHR mutations have been shown to compromise IN-RAL interactions but not those between IN and DTG or IN and EVG [166]. The dissociative *t*_1/2_ values range between 42–60 h for DTG, 1.1–2.0 h for RAL, and 1.6–2.1 h for EVG compared to EC_50_ FC ranges of 0.89–1.4, 3.2–16 and 1.5–1.8 for DTG, RAL, and EVG, respectively [164]. The notion of a relationship between dissociative *t*_1/2_ and antiviral potency is further supported by data on the E92Q/N155H, E138K/Q148R, and G140S/Q148R substitutions in IN [164]. 

DTG is being investigated in INI-salvage therapy in an ongoing study termed VIKING. This is a phase II single arm study investigating the feasibility of replacing RAL with DTG in patients experiencing failure due to RAL-resistant viruses [151]. Participants (*n* = 27) were switched from their previous RAL-containing regimens to receive 50 mg once daily DTG for ten days and were then prescribed other active drugs over a period of 23 weeks. Eighteen of the study participants had INI- resistant viruses belonging to the Y143, Q148 and N155 pathways prior to initiation of the study. After 10 days of DTG monotherapy, all participants harboring viruses in the Y143 and N155 pathways attained a mean HIV-1 RNA decrease of *≈*1.8 log copies/mL compared to *≈*0.7 log copies/mL for viruses harboring G140S/Q148HKR double mutations. None of the viruses harboring Q148HRK plus two or more additional mutations experienced a decrease of ≥0.7 log copies/mL, indicating a degree of resistance on the part of Q148HKR viruses to DTG. This trial nonetheless provided proof-of-principle for the use of DTG in RAL-experienced patients infected by subtype-B viruses harboring mutations at position Y143 and N155.

Several serial passaging studies have been carried out in order to model the effects of DTG in RAL experienced patients and showed that the presence of the N155H and Y143C/H/R resistance did not lead to development of additional resistance mutations under DTG pressure nor to a substantial decrease in DTG susceptibility [4, 29]. In contrast, the presence of Q148HKR mutations did lead to further mutations and >100 FC for DTG susceptibility relative to wild-type in subtype B viruses [155, 157]. Interestingly, Q148HKR mutations did not affect susceptibility to DTG in HIV-1 subtype C and HIV-2 isolates [157, 167, 168]. An ongoing trial termed SPRING-2 will evaluate the use of once daily DTG versus twice daily RAL in treatment-naïve patients. A Phase III trial termed SAILING will compare once-daily versus twice-daily DTG in ARV-experienced INI-naïve HIV-positive subjects [169]. 

#### 3.3.3. S/GSK-1265744

This compound is another second generation INSTI and is a back-up drug to DTG that has been tested in double-blind randomized placebo-controlled trials (Table 3) and has shown promising short-term efficacy, an excellent pharmacokinetic profile, and good tolerability in HIV-positive patients [170]. Its future development is uncertain, given the positive state of development and promise of DTG that is now in advanced phase 3 clinical trials.

### 3.4. Integrase Structural Studies

The study of quantitative structure-property/activity relationships (QSPR/QSAR) has led to major breakthroughs. Recent elucidation of the full-length prototype foamy virus (PFV) structure [171], intasome [172] and strand transfer complexes (STC) [166, 173, 174] with DNA ligands, and subsequently with RAL, DTG, and EVG and MK-2048 [166, 172], have allowed for the generation of homology models of HIV-IN that can be used to “train” and score drug prototypes. There are multiple QSPR/QSAR protocols and programs, some of which require advanced programming and mathematical skills, but several stand-alone and online programs offer semiautomated drug docking and scoring capabilities with moderate to high accuracy. The main aim of these approaches is to allow *in silico* validation and testing of prototype molecules in order to lower the costs associated with large-scale synthesis of non-validated compounds [175]. Typical QSPR/QSAR protocols use a given set of conditions that “train”/test the structures and a set of validatory parameters that are then used to score the data. Structures can then be selected for subsequent synthesis and experimental validation [176]. Typical input takes into account the physicochemical properties of individual moieties on the compound, bond-length, flexibility, lipophilicity/hydrophilicity, information on the target and 3-dimensional binding space. This can generate theoretical estimations of IC_50_, binding affinity, bioavailability, hepatic clearance, and other parameters. A recent summary of computer-based approaches for design of novel INIs that target 3′ processing, IN multimerization, strand transfer complex assembly, and IN-host protein interactions has been published [177]. Despite these advances, it is difficult to accurately model drug toxicity, bioavailability, and safety prior to the synthesis and study of novel candidate drugs. 

### 3.5. Newer Strand Transfer Inhibitors in Preclinical Development

MK-0536 was synthesized by Merck & Co., based on quantitative structure-property/activity relationships (QSPR/QSAR) that took into account the optimum minimum structure necessary for activity and generated a set of potential structures that could be synthesized and screened. MK-0536 has low hepatocyte clearance values [178] and generally good inhibition of wild-type IN and RAL resistant IN [178, 179] but its current level of clinical development is unclear. Other classes of compounds that block strand transfer with high specificity at subnanomolar EC_50_s and low toxicities are cathechol-based [180], pyrimidone-based [181–184], dihydroxypyrido-pyrazine-1,6-diones [185], and quinolones [186, 187] among others.

### 3.6. Blocking IN-Host Interactions

The fact that IN takes part in a number of interactions with host proteins and their posttranslational modifications has led to the targeting of some of these processes (reviewed in [188, 189]). Separate studies have shown that sumoylation [190] and acetylation [191] of IN occurs *in vivo,* leading to increased activity, but there are currently no specific inhibitors of these reactions that can specifically target IN modification without affecting other cellular proteins [192]. The observation that integration can be inhibited in *ex vivo *HIV-infected CD4^+^ T cells of elite controllers [192] has not yet led to the identification of a responsible cellular factor. Currently, the most promising inhibitors targeting IN-host interactions disrupt interaction between IN and LEDGF/p75; the latter is a host protein that has been shown to be essential for tethering the IN preintegration complex (PIC) to host chromatin and also for the recruitment of other cellular factors to the PIC, thereby facilitating effective integration [193, 194]. Integration cannot occur in the absence of LEDGF/p75 and inhibition of the LEDGF/p75-IN interaction can block viral replication [193, 195]. This is supported by the recent finding that polymorphisms in the PSIP-1 gene that codes for LEDGF/p75 can affect progression of HIV disease [196].

#### 3.6.1. The LEDGF/p75-IN Interaction

The compounds termed LEDGINS were designed as specific small molecular inhibitors of the LEDGF/p75 interaction. 2-(quinolin-3-yl) acetic acid derivatives have been cocrystallized with LEDGF/p75-IN and optimized structures within this group were shown to inhibit the LEDGF/p75-IN interaction at sub *μ*M concentrations [193, 195]. Peptides mimicking the IN-binding domain (IBD) of LEDGF/p75 exhibit potent inhibition of IN [197, 198]. A number of reviews on the subject of LEDGF/p75 targeted INIs have been published [195, 198, 199].

### 3.7. Inhibitors of 3′ Processing

#### 3.7.1. BI 224436

This compound is a novel INI with a distinct mode of action from more established INSTIs. It is a noncatalytic site integrase inhibitor (NCINI) (Table 3) which interferes with the interaction between IN and the chromatin targeting the LEDGF/p75 protein, yielding low nM inhibition of 3′ processing and viruses [200]. The profile of this compound appears favorable and it also appears to be specific since it did not exhibit reduced activity against any INSTI-resistant IN enzymes [200]. This compound has now entered phase Ia clinical trials to evaluate dosing and safety in healthy individuals. Initial reports indicate high bioavailability with good tolerability at single doses ranging up to 200 mg. BI 224436 also exhibited good-dose-proportional pharmacokinetics when given as a single-dose of 100 mg, and plasma levels appeared adequate to achieve a positive result [201]. 

### 3.8. Dual RT/IN Inhibitors

Structural and functional similarities between HIV-1 IN and the RNAse-H domain of HIV-1 RT suggest the possibility of specific yet dual targeting inhibitors of both processes. Some early compounds that have been found to target both enzymes are diketo acids (DKA) [202, 203]. This hybrid class has been reviewed [204]. 

### 3.9. Integrase Inhibitors and HIV Diversity

Subtype differences may exist in regard to development of resistance to IN inhibitors, a phenomenon that also exists with RT inhibitors [119, 205, 206]. Despite the fact that HIV-1 subtype B and C wild-type IN enzymes are similarly susceptible to clinically validated INIs [153], the presence of resistance mutations may differentially affect susceptibility to specific INSTIs [119]. Recent reports suggest that the G118R mutation, which was previously reported to confer slight resistance to MK-2048, imparts a 25-fold resistance to RAL when present together with the polymorphic mutation L74 M in CRF-AG cloned patient isolates [207]. Additionally, it is well documented that the INI Q148RHK resistance mutations, which affect susceptibility to DTG in HIV-1 subtype B, may not affect the susceptibility of either HIV-1 subtype C or HIV-2 enzymes to DTG and other INSTIs [164].

### 3.10. Resistance to INSTIs

As indicated above, drug resistance threatens the long-term efficacy of HAART. The problem of resistance in HIV therapy has been reviewed elsewhere [208, 209]. Against this background, the addition of new classes of drugs is important to limit the emergence of resistant strains. 

Integration is a well-known two-step reaction catalyzed by the retroviral protein integrase [210, 211]. Two distinct reactions (3′ processing and strand transfer) require the coordination of divalent ions (Mg^2+^ or Mn^2+^) by a catalytic triad consisting of amino acids D64, D116, and E152. INSTIs bind to integrase and specifically target the strand transfer step of integration [212, 213]. These drugs have demonstrated their efficacy against HIV *in vitro* and in patients and are particularly useful against viral strains that are resistant to other drug classes [214]. They are active against both B- and non-B HIV subtypes both in tissue culture and in patients [49, 215]. The prototypic INSTI, raltegravir (RAL) [216, 217], was approved for therapy in 2007, while elvitegravir (EVG) [218] and dolutegravir (DTG) [219] are now in advanced clinical trials. Resistance pathways against first-generation INSTIs were identified whose primary mutations include the substitutions N155H, Q148 K/R/H, and Y143R/C [215, 220–222]. Second-generation INSTIs, such as MK-2048 [223–226] and DTG [223, 225–230], possess a more robust resistance profile than RAL and EVG [225, 226, 230–232]. Several other compounds are currently in early development, such as compound G [226].

### 3.11. Mutations against Newer INSTIs

Several resistance mutations have been identified in regard to second-generation INSTIs. Although some of these mutations were previously observed with first-generation drugs, most of them are still poorly characterized. Initial *in vitro* selection studies with DTG have led to the identification of mutations T124A, S153F/Y, and L101I [230, 233]. However, experiments with viruses containing these mutations showed that none of them, alone or in combination, significantly decreased DTG susceptibility. When similar selections were undertaken with viruses containing either Q148R or Q148H at baseline, numerous additional mutations were identified, among which E138K and G140S were the most common [233]. Importantly, even the combination of five of these mutations did not decrease drug susceptibility to DTG by more than approximately 20 fold in this study [233]. In other *in vitro *selection experiments, additional mutations were identified in subtype B, C, and recombinant A/G viruses [234]. The R263K mutation was the most common mutation identified in this last study and conferred low-level resistance to DTG as well as a decrease in viral fitness and strand transfer activity *in vitro*, due to a reduction in DNA binding [234]. Additional mutations H51Y, S153Y, and G118R were identified in the same study [234]. Similar *in vitro *selection studies with MK-2048 led to the appearance of G118R and the secondary mutations E138K [225], G140E [231], N155H, and S153Y [223]. 

### 3.12. Mode of Action

**Figure 1:**
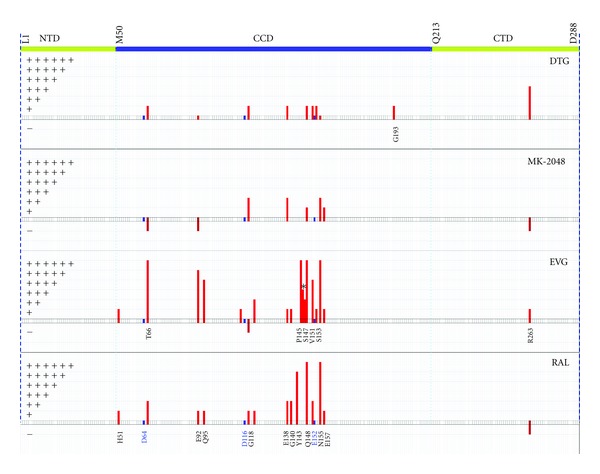
Resistance map of HIV-1 integrase showing residues prone to mutate into resistance mutations and associated levels of resistance/susceptibility. For clarity, each position associated with resistance is labelled only once and the drug that was first reported to select for a particular mutation is stipulated. Key active site residues are indicated in blue. Levels of resistance shown in the figure as indicated by fold-change (FC) relative to wild-type are as follows: FC values < 1 are designated (−) and those between 1–3, 3–5, 5–10, 10–15, 15–20, and >20 are designated +, ++, +++, ++++, +++++, and ++++++, respectively. N-terminal domain (NTD), catatlytic core domain (CCD), C-terminal domain (CTD). Data were obtained from [223, 225, 226, 230, 232, 234, 246–250] as well as from the Stanford HIV Drug Resistance Database [251].

Resolution of the structure of the prototype foamy virus (PFV) intasome has been broadly used to explore the mechanisms of action of INSTIs and their resistance mutations [213, 235–238]. INSTIs bind to the catalytic core domain of integrase and compete with host DNA binding [239]. These drugs contain a halogenated phenyl group that invades the catalytic pocket and displaces the 3′ viral end [236, 240] and three coplanar oxygen atoms that chelate the divalent ions within the catalytic pocket, thus inhibiting the activity of the catalytic DDE triad [211, 213, 232, 241–243]. Although coordination of these ions by the triad is also necessary for 3′ processing, INSTIs are specific for strand transfer and only poorly inhibit 3′ processing [212, 239]. This is due to an allosteric hindrance between the halogenated phenyl group and the 3′ dinucleotide cleaved during 3′ processing that prevents efficient binding of INSTIs before 3′ processing occurs [236, 240]. The fact that all early integrase inhibitors target the catalytic core domain is an argument for the development of alternative strategies to block integration, such as allosteric integrase inhibitors (ALLINIs). Not surprisingly, most INSTI resistance mutations lie in the catalytic core domain surrounding the catalytic pocket that contains the DDE triad (Figure 1). Y143 is located within the active site, and D64 and E152 surround N155, and Q148 lies at the core of the triad, in close proximity with all three acidic residues of the triad. In addition, Q148 interacts with the viral DNA 5′ end [244]. The G118 residue is highly conserved, and is in close proximity with a key residue of the triad, D116. Similarly, S153 is next to E152. In contrast, R263K is an unusual mutation at the C-terminal end of the protein. R263K is not within the catalytic pocket but contributes to viral DNA binding [234] and might be important for nuclear import of integrase [245].

### 3.13. Mechanisms of Resistance

The strategic locations of the Q148H and N155H mutations are thought to play a role in triggering conformational changes within the catalytic pocket that result in an increase in the binding energy of INSTIs [240]. In contrast to these two residues that do not make direct contact with INSTIs, Y143 is thought to connect to RAL by pi stacking between the tyrosine phenol ring and the RAL 1,3,4 oxadiazole group [240]. EVG, MK-2048 and DTG do not have this oxadiazole group and are largely resistant to mutations at position Y143 [226, 246]. G118R may cause changes in the geometry of the catalytic triad, seemingly decreasing the ability of second-generation INSTIs to chelate divalent ions within the catalytic core [225]. 

Alternatively, this mutation could decrease drug binding by steric hindrance [232]. The mechanism of resistance of the DTG-associated resistance mutation R263K is not well characterized but could be linked to decreases in viral DNA binding, as described previously [234]. Important results have emerged through measurements of the RAL, EVG and DTG dissociation rate constants [252] from an integrase-DNA complex containing integrase proteins bearing resistance mutations. These studies demonstrated a striking correlation between dissociation rate and levels of resistance [253]. Both the Q148H and N155H resistance mutations increased the DTG *k*_*off*⁡_ by 13.7 and 7.4 fold, respectively, whereas the Y143 mutations had little effect [253]. The same study showed that Q148H and N155H both significantly increased the *k*_*off*⁡_ for RAL and EVG, but that Y143C/H/R affected only RAL but not EVG dissociation from IN-DNA [253]. This result is in agreement with the absence of resistance to EVG by the Y143R mutation [246].

This suggests that the three main pathways of resistance to INSTIs act, at least in part, by reducing the residence time of the drugs within integrase. Indeed the possible association between slow dissociation rate and the development of resistance has been documented in other studies [253, 254]. Dissociation rate is therefore an important factor measuring the quality of a drug [254] and should be taken into account in the development of future integrase inhibitors, since INSTIs bind with high affinity to the integrase enzyme and dissociation is a slow process that is sometimes almost irreversible. Since the integrase enzyme is packaged within the viral particle and is delivered to the infected cell as a single dose, this excludes that new integrase target structures will be synthesized prior to integration. However, resistance mutations that alter integrase-DNA binding activity, stability or localization of the preintegration complex might impact the activity of the INSTIs. Further investigation of this subject is needed.

### 3.14. Resistance Testing

Testing for resistance is studied phenotypically in cell culture or biochemically using recombinant purified integrase proteins. These two approaches commonly yield comparable levels of resistance. However, this is not always the case [234], suggesting that nuclear import, stability of the preintegration complex and/or host factors could play a role in resistance to INSTIs. Cell culture resistance is usually measured during a single-cycle of replication within cells expressing either luciferase (Luc) or green fluorescent protein (GFP), under the control of a HIV long terminal repeat [255] promoter. Biochemical resistance can be measured using radioactive- or fluorescence-based assays performed on gels or plates as well as by other methods. 

Drug resistance can be tested in tissue culture selection studies by sequencing the integrase region of the *pol* gene of the viral genome after increasing drug concentration. Alternatively, viruses that are obtained from patients can be sequenced after treatment failure. There is usually a good correlation between mutations selected in cultureand those detected in patients. In preclinical studies, tissue culture selections are performed by infecting cells in the presence of suboptimal concentration of inhibitor [256]. The choice of cells used during these selections can impact on the development of resistance mutations. For example, selection with DTG in MT-2 cells resulted in the emergence of T124A, S153F/Y and L101I in subtype B virus whereas similar experiments in cord blood monocytic cells (CBMCs) yielded the R263K mutation [234]. The stochastic emergence of resistance mutations can sometimes also be demonstrated [231, 247, 257, 258]. In addition, no study has demonstrated that a particular cell model is better than others for selecting clinically relevant resistance mutations. This underlines the importance of performing multiple *in vitro *selection studies using different types of cells. 

Ultradeep sequencing and other novel methods to assess the development of resistance against integrase inhibitors in patients are currently being developed [259]. Recently, 23 patients receiving RAL-based salvage therapy were monitored longitudinally for the emergence of resistance mutations [260]. This study confirmed the role of the three main resistance pathways Y143, N155, and Q148 for RAL resistance and the potency of ultradeep sequencing [259]. Studies on the evolution of resistance mutations in patients infected with viral strains resistant to multiple classes of drugs and treated with EVG showed that integrase resistance mutations identified during early treatment failure were often absent from later time points because they had been substituted by other mutations [261]. Similar observations have been made with RAL [259, 262–264] and the development of resistance through the cumulative acquisition of mutations has also been described for this drug [259, 260, 262–264]. Thus, longitudinal genotyping of viral samples from patients undergoing integrase inhibitor treatment should be implemented when possible. This will improve our understanding of the emergence and dynamics of resistance mutations associated with INSTIs. In regard to both RAL and EVG, Q148 mutations can emerge and substitute for initial mutations at Y143 and N155 [223, 247, 259, 261, 265]. The emergence of Q148 in the presence of alternative resistance pathways seems due to increased viral replication fitness associated with the latter mutation and is of concern in regard to potential cross-resistance against INSTIs of both first and later generations. 

### 3.15. Cross-Resistance

Newer inhibitors have been developed to avoid cross-resistance with first-generation drugs, but bind to the same catalytic site and possess similar chemical properties as first-generation INSTIs. Multiple cross-resistance mutations have been described for the first-generation drugs RAL and EVG (reviewed in [248, 266]). Among the three main resistance pathways, a notable exception is Y143H/R which is specific for RAL and does not compromise the activity of EVG [246]. However, the addition of other secondary mutations to Y143 mutations can confer up to 66-fold resistance to EVG [226]. The two other pathways, N155 and Q148, confer cross-resistance against both EVG and RAL [248, 266]. 

G118R was originally identified during tissue culture selection studies with the second-generation INSTI MK-2048 [225] and was later identified in a patient with a CRF02_AG infection who failed RAL treatment [207]. In addition, G118R was shown to confer resistance to MK-2048, RAL and DTG [207, 225, 232]. The reason that G118R remains uncommon in patients treated with RAL might be its severe impact on viral fitness [225]. The secondary mutation E138K that augments levels of MK-2048 resistance only partially restores viral fitness in viruses that contain G118R [225]. S153Y is another mutation that confers low-level cross-resistance to both MK-2048 and DTG [223, 230, 233, 234, 267]. Another second-generation INSTI resistance mutation, R263K, was originally identified as a secondary mutation to T66I during selection studies with EVG and R263K confers ~5-fold resistance against EVG [257, 268] but not against RAL [268], as was also observed in a biochemical strand transfer assay [234]. Thus, some degree of cross-resistance exists between the first- and second-generation INSTIs, but most of the mutations responsible seem to confer only low- or modest-level resistance to the second-generation drugs. 

In contrast, mutations emerging from the Q148 pathway seem to be more potent against the latter drugs and Q148K/E138K, Q148R/N155H, and Q148R/G140S confer 19-, 10-, and 8-fold resistance, respectively, to DTG [230]. The addition of further secondary mutations within the Q148HR/G140S pathway can lead to as much as 27-fold resistance to DTG [269]. Similarly, the Q148H/G140S mutations can decrease susceptibility to MK-2048 by 12-fold [226] and mutations at position Q148 with multiple secondary mutations can result in up to 250-fold resistance to this drug [223, 231]. These data indicate that mutations that emerge from the Q148 pathway can confer cross-resistance to second-generation INSTIs, in contrast with the Y143 and N155 mutational pathways [226]. 

### 3.16. Other Findings

Selection studies in tissue culture have identified several mutations that reduce HIV-1 susceptibility to DTG and MK-2048. Resistance pathways involving mutations at positions G118, R263, S153, N155, and Q148 can limit second-generation INSTI activity. However, only resistance mutations emerging from the Q148 pathway seem to confer a significant (i.e., >10-fold) decrease in susceptibility to these compounds. Importantly, second-generation INSTIs have proven to be robust against the emergence of resistance, as shown in the phase IIb SPRING clinical studies with DTG, for which no resistance mutation has yet been identified in patients after 96 weeks of treatment [270, 271]. In addition, DTG has been shown to be active against viruses resistant to RAL [272, 273], although viruses bearing Q148 mutations together with several secondary mutations can necessitate an increase in the clinical dose of DTG [272, 273]. Clearly, the hope is that second-generation INSTIs will be able to target viruses that are resistant to first-generation drugs. The emergence of mutations at position Q148 should be monitored carefully during therapy due to the possible emergence of novel resistance pathways. Alternative strategies that target integration but that are not based on the catalytic triad, for example, allosteric integrase inhibitors (ALLINIs), are promising and such compounds retain full activity against viruses that are resistant to INSTIs. 

## 4. Conclusion

New HIV inhibitors should ideally possess a high genetic barrier in regard to potential development of resistance for this same drug class as well as a unique resistance profile in both B and HIV-1 non-B subtypes. NRTI resistance mutations are widespread and there is currently no approved NNRTI compound that possesses activity against all NRTI mutations. However, a number of new NRTIs with potent activity against NRTI-resistant viruses are now in preclinical and clinical development. In some cases, these compounds possess the ability to maintain high concentrations inside cells (e.g., CMX157, GS-9131, and GS-7340) or may have a different mechanism of action than older NRTIs, that seems to be the case for EFdA-TP. Although M184V is a common NRTI mutation high-level resistance to 3TC and FTC and cross-resistance to other NRTIs, the levels of resistance conferred against newer NRTIs (ELV, ATC, and EFdA-TP), is low-level, suggesting that these compounds may be useful against M184V-containing viruses. It will be important to determine whether selection of M184V in patients receiving ELV, ATC or EFdA-TP in elevated viral loads and treatment failure. The potential of these novel compounds to synergize either together or with currently approved drugs will make them important components of future antiretroviral treatment. 

 The only NNRTI approved for treatment of HIV-1 NNRTI experienced patients is ETR. In a limited number of studies, ETR has proven to be effective in patients with treatment failure who harbor viruses that are resistant to initial NNRTIs and that carry several resistance mutations. A number of studies have shown that patients who fail NVP therapy are more prone to develop ETR resistance mutations faster than those who fail EFV therapy. Considering the wide-spread use of NVP in developing countries, the use of ETR in NNRTI experienced patients in these settings is under debate. RPV that is now approved for treatment naïve patients shows high level cross-resistance with ETR. Therefore, the use of RPV in first-line therapy may jeopardize the future of ETR as a second line NNRTI and the sequential use of these drugs is not recommended. The emergence of E138 K as a signature mutation for almost all new NNRTIs (both approved and under clinical development) is another limitation of these drugs. 

RDEA806, lersivirine and TMC120 are still in phase II clinical trial and large scale phase III trials are required to exploit their potential use in the clinics. Some mutations have been selected in the presence of these three compounds together with ETR and RPV. The fact that TMC120 in combination with TFV as a microbicide is more potent and decreases the possibility of selecting for resistance mutations than either compound alone shows potential for such an antiviral based microbicide in preventing HIV-1 infection. The search for novel NNRTIs should now focus on compounds with different resistance profiles, so as to broaden treatment options for patients who have experienced NNRTI therapy failure. Overall, new NRTI and NNRTI agents can provide a welcome therapy option for patients with existing NRTI or NNRTI resistance and also for patients who have never received treatment with ARVs. 

INSTIs represent a new drug class in the anti-HIV armamentarium. New compounds are being developed that possess improved resistance profiles and pharmacokinetics. It therefore seems likely that INSTIs will be a future stalwart of antiretroviral therapy. These advances have been accompanied by improved understanding of integrase function which, in turn, is leading to identification of new molecules that can block integrase function through novel approaches.

## Figures and Tables

**Table 1:** New NRTIs that are approved or are undergoing clinical development.

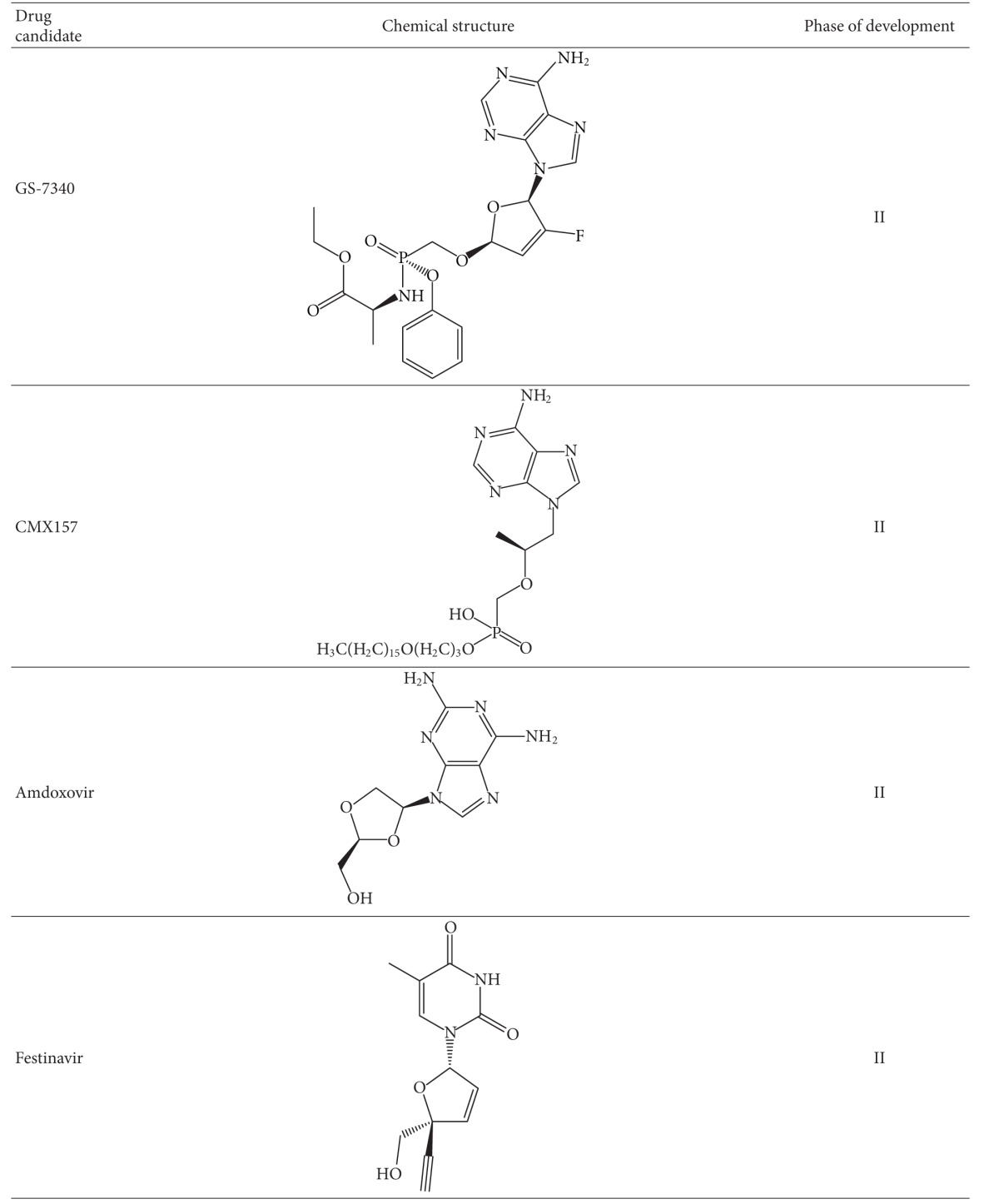 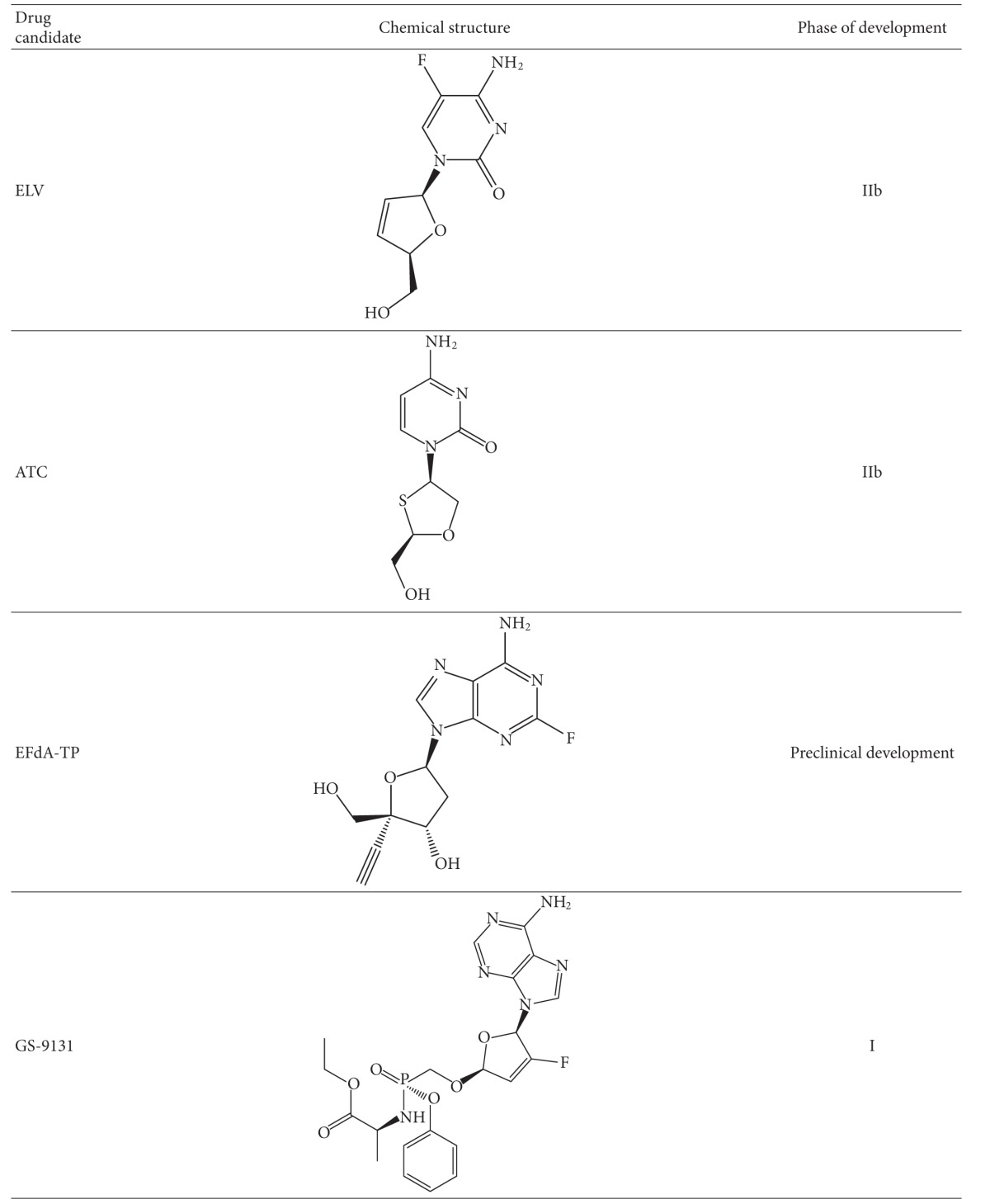

**Table 2:** New NNRTIs that are approved or are undergoing clinical development.

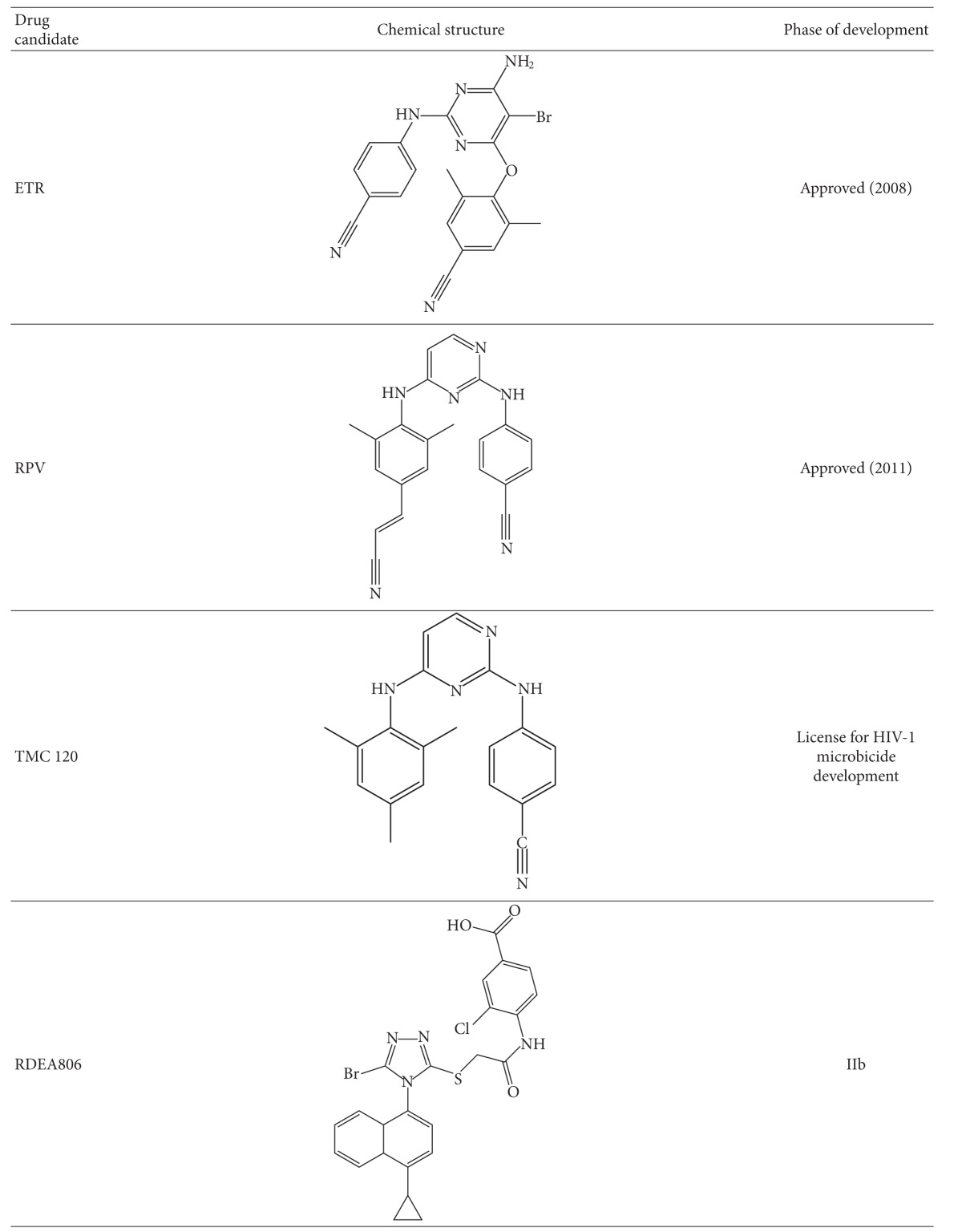 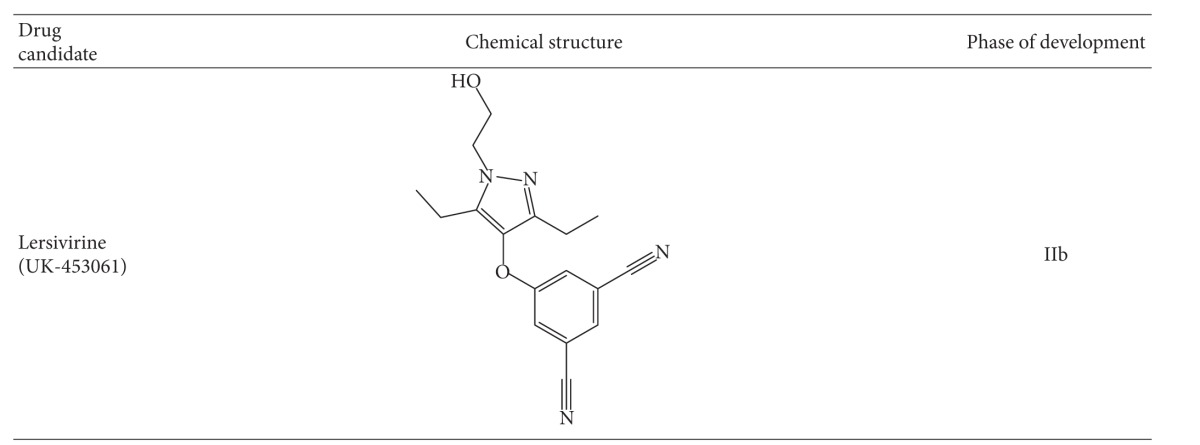

**Table 3:** Clinically relevant INI compound structures.